# A Review on Parrot Gut Health: Insights into the Intestinal Structure, Microbiota Diversity, Influencing Factors, and Nutritional Strategies

**DOI:** 10.3390/vetsci13101079

**Published:** 2026-10-12

**Authors:** Yan Jia, Yan Li, Aftab Shaukat, Shu-Cheng Huang

**Affiliations:** 1 School of Animal Engineering, Xuzhou Vocational College of Bioengineering, Xuzhou 221006, China; jiayan15248140310@163.com; 2 College of Pet Science and Technology, Zhengzhou Urban Construction Vocational College, Zhengzhou 451200, China; dapangtu2026@163.com; 3 College of Veterinary Medicine, South China Agricultural University, Guangzhou 510642, China; dr.aftabshaukat@scau.edu.cn; 4 College of Veterinary Medicine, Henan Agricultural University, Zhengzhou 450046, China

**Keywords:** bird, gut microbiota, nutritional regulation, One Health, probiotics, pet, trace elements

## Abstract

The parrots, a highly intelligent avian species, possesses distinctive characteristics including remarkable cognitive abilities, strong mimetic capacity, and significant economic value as companion pets, with the scale of commercial captive breeding expanding considerably over recent decades. Intestinal health is critical for nutrient digestion and absorption, and serves as a primary barrier against pathogen invasion; thus, maintaining optimal gut function is essential for enhancing overall health and breeding performance. However, compared to other poultry species, research on the pivotal role of gut health in the breeding and management of parrots remains limited. This review summarizes the unique anatomical and physiological features of the parrot digestive system, the characteristics of its gut microbiota, and their interrelationship with intestinal health, and further discusses the significance of intestinal health for the overall well-being of parrots. Additionally, this review highlights emerging nutritional regulation strategies, particularly the application of probiotics and other functional additives for improving intestinal health, which will provide new insights into enhancing the health status of parrot populations and formulating management strategies for the parrot breeding industry.

## Simple Summary

Parrots are highly valued ornamental birds mainly raised as companion pets, and the volume and scale of their commercial captive breeding have grown dramatically in recent decades. The intestine is an important site for nutrient absorption and digestion, and also acts as a key barrier against pathogen invasion. However, the pivotal role of gut health in the healthy breeding and management of parrots has not been sufficiently emphasized. Therefore, this review summarizes the unique anatomical and physiological features of the parrot digestive system, the characteristics of its gut microbiota, and their interrelationship with intestinal health, and further discusses the significance of intestinal health for the overall well-being of parrots. Additionally, this review highlights emerging nutritional regulation strategies, particularly the application of probiotics and other functional additives for improving intestinal health, which may provide new insights into enhancing the health status of parrot populations and formulating management strategies for the parrot breeding industry.

## 1. Introduction

### 1.1. Breeding Status and Health Risks

Currently, the global consumer demand for ornamental parrots is on a steady upward trend. Data from the “Global Bird Pet Market Report” indicates that parrots have emerged as the dominant segment within the pet bird market, followed by Passeriformes (passerines), Falconiformes (falcons) [1]. One of the three families of true parrots, Psittacidae, was among the twenty most heavily traded families in the global bird trade [2]. However, the rapid expansion of breeding scale is often accompanied by a heightened risk of disease transmission; notably, viral and parasitic intestinal diseases pose primary threats to parrot health.

Parrot bornavirus (PaBV), a highly pathogenic virus prevalent in captive parrot populations, induces proventricular dilatation disease (PDD). Infected parrots exhibit severe digestive disorders, including proventricular dilatation, food retention, emaciation, and diarrhoea, accompanied by neurological symptoms such as ataxia, convulsions, and behavioral abnormalities [3,4]. Epidemiological surveys indicate high infection rates of this virus within the investigated clinical or farm-specific cohorts, with reported prevalences of 45.97% in Taiwan, China, and up to 73.7% on some farms in Brazil [3,4]. These prevalence estimates are based on selected sampling cohorts and cannot be generalized to all captive parrot populations, although PaBV infection is associated with elevated mortality risk among affected individuals within these studied populations. Furthermore, in the artificial breeding of endangered parrots, such as the Puerto Rican parrot, the captive environment is prone to inducing nutritional imbalances. As natural omnivorous birds, parrots require the ingestion of special foods, such as branches, leaves, and minerals, to maintain optimal intestinal physiological functions. However, captive breeding practices often rely on monotonous diets, such as pure seeds. This directly leads to slowed intestinal peristalsis and reduced gut microbiota diversity, thereby elevating health risks [5,6]. Collectively, these issues demonstrate the urgency of implementing effective intestinal health management for parrots within both the breeding industry and endangered species conservation sectors.

### 1.2. The Roles of Intestinal Health in Parrots

The intestine serves not only as the primary site for nutrient digestion and absorption but also as the largest immune organ, playing an irreplaceable key role in maintaining overall health [7,8]. For parrots, intestinal health is closely associated with growth rates, reproductive performance, and disease resistance [5]. Wild parrots maintain microbial balance through diverse diets, supplementing nutrients via the ingestion of invertebrates and natural minerals. Conversely, monotonous diets under captive conditions frequently lead to trace element deficiencies and diminished gut microbiota diversity, which can trigger intestinal inflammation and metabolic disorders [5,6,9]. The dietary structure of wild parrots may provide valuable clues: the diet of captive parrots should be more diverse to preserve gut microbiota diversity.

The composition and function of the gut microbiota are crucial for host health [7,10,11]. Fecal microbiome analysis of Puerto Rican parrots reveals a coexistence of bacteria (96%), viruses (3%), and eukaryotes (0.6%) in the gut, where the primary functions of the microbiota involve carbohydrate metabolism (14%) and protein metabolism (8%) [12]. Moreover, a direct correlation exists between gut microbiota disorders and digestive diseases [5,13]. Notably, the gut microbiota of captive parrots exhibits low stability and is highly susceptible to dietary fluctuations [5]. Once intestinal function is compromised, it is associated with the emergence of a series of health problems, such as indigestion, diarrhea, and decreased immunity, which may even be life-threatening in severe cases.

In recent years, research on parrot intestinal health has advanced significantly, progressing from fundamental insights to practical application, with notable breakthroughs in the analysis of gut microbiota composition and the exploration of probiotic applications [14,15]. For example, studies on gastrointestinal microbiota have clarified significant structural differences across different digestive tract segments of birds, such as the crop and small intestine. The state of hindgut microbiota can be well reflected by fecal samples, providing a scientific basis for non-invasive monitoring of intestinal health [16]. Additionally, probiotic interventions have demonstrated significant potential in enhancing gut microbiota homeostasis and digestive efficiency [8,14,15,17,18]. However, research on parrot intestinal health lags behind that of poultry and other common avian species, exhibiting two primary deficiencies. First, research objects have predominantly focused on common pet parrots, such as cockatiels and budgerigars, with a relative lack of research regarding the gut microbiota diversity characteristics and special nutritional needs of wild parrots, such as the Puerto Rican parrots. Second, a systematic and comprehensive evaluation system for parrot intestinal health has yet to be established [14]. Therefore, this paper aims to systematically integrate existing research results and analyze parrot intestinal structural characteristics alongside intervention measures, thereby providing a theoretical reference for the in-depth study of parrot intestinal health.

## 2. Intestinal Anatomical and Physiological Characteristics of Parrots

### 2.1. Composition of the Digestive System

To adapt to their unique feeding habits, the parrot digestive system has evolved a series of specialized structures different from those of mammals and other avian species. The complete digestive tract consists of the oropharynx, esophagus, crop, proventriculus (glandular stomach), gizzard (muscular stomach), small intestine, cecum, rectum, and cloaca. Each segment performs distinct functional roles in the digestive process, collectively ensuring the efficiency of nutrient acquisition (Table 1).

#### 2.1.1. Crop

The crop is a specialized distal structure of the esophagus with robust expansion capacity, serving primarily for the temporary storage of food. This adaptation allows parrots to ingest large amounts of food rapidly and subsequently transport it to the stomach via rhythmic contractions, effectively supporting their survival needs for swift foraging and predator avoidance in the wild [19]. Additionally, the crop in some parrot species (e.g., *Anodorhynchus hyacinthinus*) produces “crop milk”, a secretion rich in lipids and proteins derived from the proliferation and shedding of lipid-laden epithelial cells. This substance serves as the exclusive initial food source for hatchlings [19]. Compared to mammalian milk, crop milk is notably deficient in carbohydrates and calcium, representing a species-specific form of nutritional supply [19]. Similar studies in pigeons have confirmed that crop milk is rich in protein and fat but contains almost no carbohydrates [26]. Although direct experimental data on parrots are limited, poultry research indicates that probiotic supplementation can improve digestive tract health, enhance nutrient absorption efficiency, accelerate crop emptying rates, and reduce the risk of diseases such as crop inflammation [8,14,27]. These findings may provide a theoretical basis for parrot breeding and management practices.

#### 2.1.2. Gizzard

The gizzard (or ventriculus), also known as the muscular stomach, is the core organ for mechanical digestion in parrots. Its walls are rich in specialized muscle tissue and lined with a tough, rough koilin layer (keratin-like membrane), often retaining grit or small pebbles within the lumen. During digestion, the gizzard undergoes powerful rhythmic contractions, the friction between the grit and the koilin layer grinds hard food, such as seeds, into fine particles, creating favorable conditions for subsequent chemical digestion and nutrient absorption in the small intestine [20,21,22]. This structure represents a compensatory adaptation to the parrot’s edentulous (toothless) physiology, enabling the efficient processing of seed-based diets [20]. Notably, the gizzard wall is composed of multiple layers of smooth muscle, and the degree of muscle development varies among species. For example, the gizzard wall of the blue-fronted Amazon parrot is relatively thin. Consequently, gizzard grinding efficiency is a critical factor, and strongly influences the digestibility and utilization rate of nutrients in the feed [20,21,22].

#### 2.1.3. Intestinal Structure

The intestinal length of parrots is typically only 2–3 times their body length, significantly shorter than that of mammals [23]. While this compact structure effectively reduces body burden during flight, it results in a shortened chyme retention time, thereby limiting nutrient absorption efficiency [22,23,24]. Compared to poultry, such as chickens, parrots possess completely degenerated ceca and a short, narrow rectum incapable of storing large amounts of feces. This anatomical constraint further weakens their ability to ferment fibrous substances [15,23,24]. Simultaneously, cecal degeneration contributes to lower gut microbiota diversity compared to birds with functional ceca. This microbiota structure is easily disturbed by dietary changes, increasing the risk of nutrient malabsorption [15,28]. Additionally, parrots lack a gallbladder and possess a limited capacity for bile concentration. This physiological trait renders them less tolerant of high-fat foods and prone to fat indigestion [22,23]. Moreover, the microbiota composition of the upper and lower digestive tracts differs significantly, and the stability of fecal microorganisms is poor, further increasing the risk of digestive system diseases [15].

### 2.2. Characteristics of Digestive Physiology

The digestive process in parrots is characterized by “high efficiency and high speed”. The volume and rate of digestive juice secretion dynamically adjust according to physiological states, such as breeding period and molting period, as well as food types. As shown in Table 2, the ingestion of high-protein or high-starch foods, such as meat and potatoes, stimulates gastrin release. This process activates calcium ion signaling pathways, thereby increasing the activity of H^+^/K^+^-ATPase in gastric parietal cells and ultimately accelerating the secretion of gastric acid and pepsin [29,30]. In contrast, when ingesting fiber-rich cereals, the secretion rate of digestive juices is significantly reduced due to the reliance on complex fermentation by gut microbiota [31,32]. This precise regulatory mechanism ensures that parrots maximize energy and nutrient acquisition efficiency under different dietary conditions.

Regarding food intake, parrots possess a thick tongue containing a spongy structure composed of muscles and connective tissues. This structure enhances the tongue’s ability to grasp and manipulate food, facilitating efficient swallowing [22]. Meanwhile, their salivary glands secrete saliva containing mucins, which not only moisten food to reduce swallowing resistance but also form a protective film around the chyme, thereby protecting the subsequent chemical digestion process [22,33].

Due to rapid intestinal transit, with food typically excreted within 2–4 h post-ingestion, high feed digestibility is considered desirable for parrots [29,30,34]. Insights from comparative avian research have put forward three potential feed-related considerations. First, animal protein may serve as a favourable protein source, with higher digestibility and absorption rates observed in other avian taxa compared with plant protein analogs. Second, avoiding resistant starch and adopting pregelatinized starch has been suggested to better match pancreatic amylase properties and improve starch utilization, although this remains insufficiently validated as a general requirement for parrots. Finally, feed requires appropriate crushing or extrusion to increase the surface area of chyme, compensating for the limited masticatory ability of parrots.

### 2.3. Intestinal Structure and Function

#### 2.3.1. Intestinal Barrier Function

The intestinal mucosa forms a triple barrier system comprising physical, chemical, and immune components, which collaboratively protect the host from pathogen invasion and damage [11]. The physical barrier consists primarily of intestinal epithelial cells and the tight junctions between them, with key tight function proteins including claudin 3, occludin, and zonula occludens-1 (ZO-1). Studies in other animal models suggest that pathogen-associated molecular patterns (PAMPs), such as lipopolysaccharide (LPS), activate inflammatory signaling pathways like NF-κB. This significantly downregulates tight junction protein expression, compromises intestinal barrier integrity, and increases mucosal permeability [11,31,35]. The defensive function of the chemical barrier relies on various substances secreted by intestinal epithelial cells. These include the mucus layer centered on mucin MUC2; digestive enzymes with hydrolytic activity, such as lysozyme; and antimicrobial peptides, such as β-defensins. These components effectively inhibit pathogen colonization on the intestinal mucosal surface through a combination of physical barrier effects and chemical sterilization [11,35,36]. The immune barrier is composed of gut-associated lymphoid tissue; immune effector cells, such as macrophages and dendritic cells; and immunologically active molecules, such as secretory immunoglobulin A (IgA). This integrated system establishes a specific immune defense line against pathogen invasion [35,36,37]. However, the specific functional characteristics and regulatory mechanisms of the intestinal barrier have not yet been verified in parrots.

#### 2.3.2. Intestinal Immune Function

The intestinal mucosal immune system facilitates pathogen clearance and regulates immune homeostasis through the secretion of various immune factors. IgA serves as the core effector molecule of mucosal immunity. Upon secretion by plasma cells in the intestinal mucosa, it combines with the secretory component to form secretory IgA (sIgA) [38]. sIgA exerts protective mucosal effects by neutralizing pathogen toxicity, blocking pathogen adhesion to intestinal epithelial cells, and regulating gut microbiota composition [37,38]. Studies have demonstrated that probiotics, such as *Bifidobacterium* and *Lactobacillus* strains, can significantly promote sIgA secretion in the intestinal mucosa and enhance overall disease resistance [37,39]. This effect is mediated through the regulation of Th1/Th2 immune balance, for example, by upregulating the expression of anti-inflammatory cytokine interleukin (IL)-10 and downregulating levels of the pro-inflammatory cytokine IL-6 [37,39]. Upon pathogen invasion, intestinal immune cells, such as T lymphocytes and macrophages, rapidly release pro-inflammatory cytokines, including tumor necrosis factor-α (TNF-α) and IL-1β, recruiting neutrophils to the infection site and activating inflammatory responses to eliminate pathogens. However, overactivated inflammatory responses can induce oxidative stress and apoptosis in intestinal tissues, ultimately leading to mucosal damage [35,40]. It is important to note that significant differences exist between birds and mammals regarding immune organ structure (e.g., the bursa of Fabricius, unique to birds), immune molecule evolution (e.g., IgY antibodies), and pathogen recognition pathways. These differences result in gaps in knowledge regarding the secretion patterns of immune factors and pathogen defense mechanisms in parrots. On the brighter side, studies on other poultry provide potential references for understanding parrots.

## 3. Composition and Function of Gut Microbiota in Parrots

### 3.1. Gut Microbiota Composition

#### 3.1.1. Gut Microbiota Structure

Studies based on 16S rRNA gene high-throughput sequencing have characterized the gut microbiota profile of parrots at the phylum level, with the core dominant microbiota including Firmicutes, Proteobacteria, and Actinobacteria [41,42]. For example, a study on nine captive parrot species revealed that Firmicutes accounted for an average of 55.5%, Proteobacteria 27.0%, Actinobacteria 10.7%, and Bacteroidetes 4.6% [41]. At the species level, parrot gut microbiota is dominated by *Lactobacillus* spp. and *Escherichia* spp. among other taxa [42,43,44]. Recently, this microbiota structure is believed to be closely associated with the relatively short intestinal anatomy of parrots and their specialized feeding habits, particularly diets based on seeds and fruits [42,44].

#### 3.1.2. Gut Microbiota Composition

There are significant species-specific differences in the composition of gut microbiota among different parrot species. For example, the relative abundance of *Lactobacillus* spp. is higher in the intestines of cockatiels, whereas the detection rate of *Escherichia (E.) coli* is significantly elevated in budgerigars [15,42,44]. These variations in microbiota composition may be associated with species-specific physiological characteristics, such as intestinal length and digestive enzyme activity, dietary preferences (e.g., seed-eating vs. frugivorous), and immune status. Consequently, these differences may contribute to apparent variations in susceptibility to intestinal diseases across different parrot species [45,46]. Additionally, cockatiels exhibit inter-individual variation in sensitivity toward specific *Lactobacillus* strains. This result suggests that inter-individual variation should be fully considered during the screening and application of probiotics for parrots to improve the safety and efficacy of probiotic interventions [15,44]. It should be noted that such observed inter-species microbiota disparities cannot be solely attributed to inherent species-specific traits. They may also be confounded by external variables including diet, age, husbandry practice, and sampling site. Therefore, caution is required when constructing generalized gut-microbiota profiles or drawing definitive species-specific microbial patterns from currently available datasets.

### 3.2. Gut Microbiota Distribution

#### 3.2.1. Distribution Differences of Gut Microbiota

The composition of gut microbiota in different segments of the avian digestive tract exhibits an obvious gradient distribution. Moreover, the upper and lower digestive tracts exhibit significant differences in microbiota composition. Notably, the microbiota composition of the respiratory tract is highly similar to that of the crop, and certain similarities are also observed among different intestinal segments [15]. In terms of specific microbiota taxa, fermentative microbiota such as *Lactobacillus* dominate the crop, while facultative anaerobes such as *Enterobacteriaceae* are the main components in the hindgut [15]. The formation of these spatial distribution patterns is closely related to the physiological environment of each segment, including pH value, oxygen concentration, digestive enzyme activity, and functional specialization. For example, the acidic environment of the crop provides suitable conditions for the colonization and proliferation of *Lactobacillus* [47]. Conversely, the hypoxic environment of the hindgut segment is more conducive to anaerobes obtaining energy through polysaccharide metabolism [48]. Both phenomena reflect the adaptive alignment between microbiota distribution and the host’s physiological environment.

#### 3.2.2. Correlation Between Fecal Samples and Gut Microbiota

Regarding sample collection methodologies for parrot gut microbiota research, fecal samples more accurately reflect the microbiota composition of the hindgut (colon and rectum) compared to cloacal swabs [12]. In contrast, the bacterial composition detected by oral swabs is more similar to that of the crop and trachea [15,49]. Further functional analysis of fecal microbiota revealed that its functional profile is highly consistent with that of the lower gut microbiota, directly validating the reliability of fecal sampling for studying the lower gut microbiota in parrots [15,44]. Functional prediction analysis indicated that the important functions of parrot gut microbiota are concentrated in carbohydrate metabolism (14%), protein metabolism (8%), and amino acid and derivative metabolism (7%). These functions may be directly involved in the host’s nutrient absorption and energy supply processes and are crucial for maintaining the normal physiological activities of birds [44]. Together, fecal samples offer practical non-invasive access to hindgut microbiota. However, it is worth noting that study outcomes may diverge partly due to differences in sample collection protocols, storage conditions, and the segment of the gastrointestinal tract effectively sampled.

### 3.3. Factors Influencing Gut Microbiota

**Figure 1:**
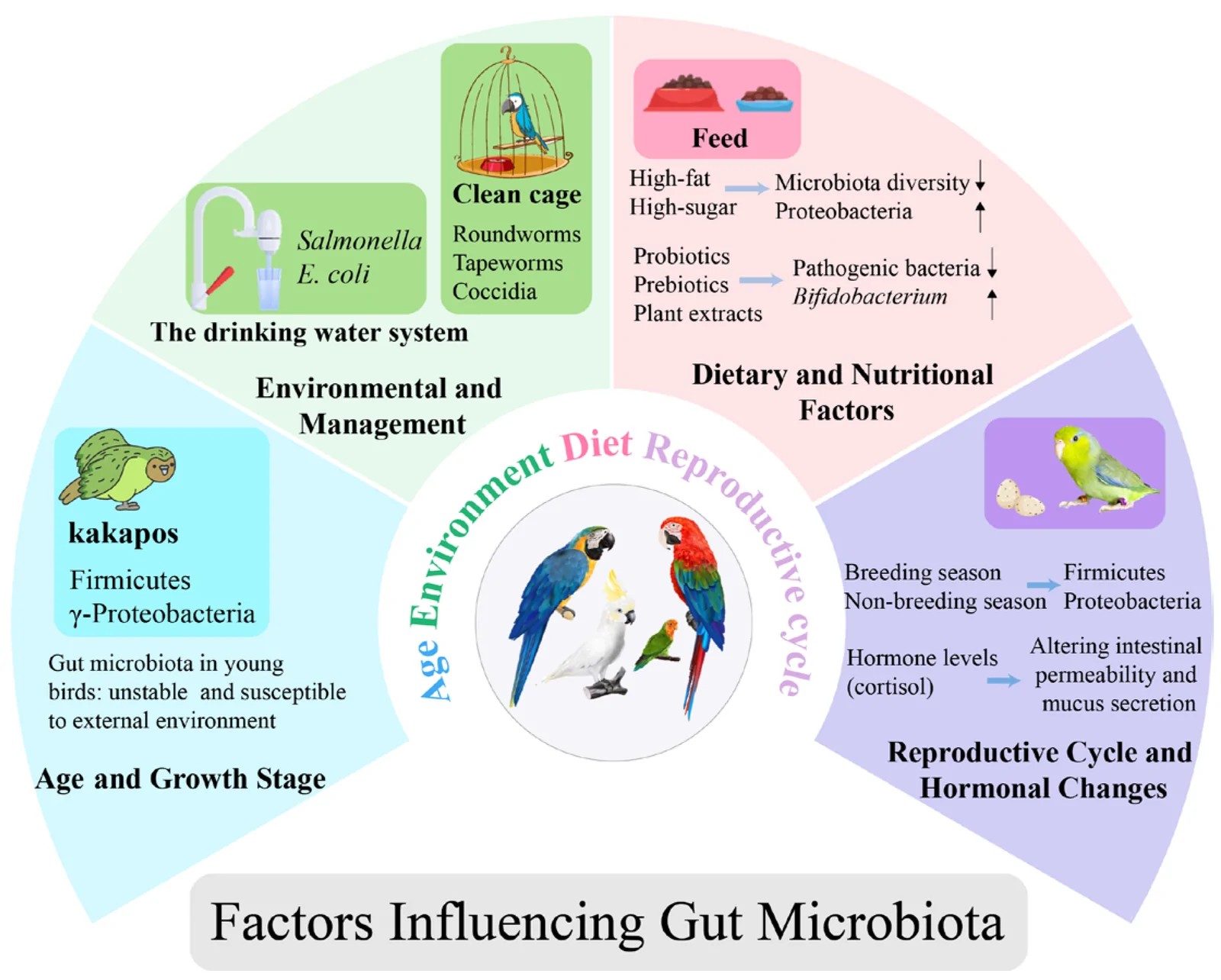
Factors influencing the gut microbiota of parrots.

The composition and function of gut microbiota are not static; rather, they are regulated by multiple factors. Dynamic changes in composition and function are closely associated with to the host’s health status, growth and development stage, and environmental adaptability [50,51,52,53,54,55]. The following sections comprehensively elaborates on the regulatory effects of various factors on parrot gut microbiota from four aspects: age, environment, diet, and reproductive cycle (Figure 1 and Table 3).

#### 3.3.1. Influence of Age and Growth Stage

Age is a key intrinsic factor affecting the composition of parrot gut microbiota. A study on kakapos found that the ratio of Firmicutes to γ-Proteobacteria is highly consistent among juvenile birds [56]. However, the gut microbiota of young birds has not yet stabilized and is significantly more sensitive to changes in the external environment than adults. This characteristic suggests that gut microbiota intervention during the juvenile stage may have a more enduring regulatory effect on their microbiota structure [41]. This “age-dependent microbiota succession” phenomenon has also been observed in other animal groups. For example, the gut microbiota of amphibian larvae (such as frogs) is dominated by Proteobacteria, whereas as individuals develop into adults, the dominant microbiota gradually shifts to Firmicutes [55,57]. In contrast, the gut microbiota of adult parrots exhibits significant individual differences. This phenomenon may be related to the mature intestinal microenvironment and fully differentiated immune system of adult individuals, which exert personalized selective pressure on the microbiota. Supporting evidence from porcine models shows that gut microbiota diversity decreases in adult animals, while the degree of functional specialization is significantly improved, including enhanced functions related to energy metabolism, confirming that age is an essential driver of gut microbiota remodeling [53,58]. In summary, the gut microbiota of parrots is characterized by a “highly conserved structure” during the juvenile stage, whereas individual differences become the main feature of the microbiota structure in adulthood.

#### 3.3.2. Influence of Environmental and Management Factors

Changes in the external environment have a significant disruptive effect on the stability of the parrot gut microbiota. Research data indicate that the oral microbiota of parrots remains relatively stable, whereas the fecal microbiota exhibits obvious instability during a 3-week environmental adaptation period. This difference directly reflects the high sensitivity of gut microbiota to environmental changes [41,59]. Regarding specific environmental risk factors, contamination of the drinking water system in the breeding environment is an essential inducer of altered microbial composition [60,61]. Pathogens such as *Salmonella* and *E. coli* can rapidly colonize open water bowls within 2 h and subsequently enter the parrot’s intestine via drinking water, leading to microbiota imbalance [60,61]. Furthermore, inadequate cage cleaning significantly increases the risk of intestinal parasitic infections [7,61]. In particular, the transmission of parasites such as roundworms, tapeworms, and coccidia can indirectly disrupt the symbiotic balance between the microbiota and host immunity, and further exacerbating gut microbiota disorders [7,61]. Notably, fecal microbiota transplantation (FMT) experiments have confirmed that supplementing beneficial intestinal bacteria such as *Lactobacillus* can effectively antagonize the colonization of pathogenic bacteria [10]. This result highlights the feasibility of improving parrot gut microbiota through external interventions [61,62].

#### 3.3.3. Influence of Dietary and Nutritional Factors

As an externally controllable factor, diet is central to regulating the composition and function of parrot gut microbiota. Long-term single feeding of high-fat, high-sugar seed diets can lead to an imbalance in the gut microbiota structure of parrots: on the one hand, it is manifested by a significant decrease in microbiota diversity; on the other hand, the proportion of Proteobacteria (especially Enterobacteriaceae) is abnormally increased, which in turn induces chronic intestinal inflammatory responses [54,63,64]. The negative impact of high-fat diets on gut microbiota has also been verified in cattle models, where such diets reduced the proportion of Bacteroidetes by 27% and increased Proteobacteria by 45%. This altered microbial composition further exacerbate metabolic disorders in the host [65]. In contrast, a diverse dietary structure can significantly improve the diversity and richness of parrot gut microbiota, especially by promoting the proliferation of short-chain fatty acid (SCFA)-producing bacteria (such as *Ruminococcus*). As vital “protective factors” for intestinal health, SCFAs can effectively enhance overall health status [64,66]. Additionally, functional components in feed, such as *Lactobacillus* in probiotics, xylooligosaccharides in prebiotics, and various plant extracts, can exert positive regulatory effects on gut microbiota. These components not only promote the proliferation of beneficial bacteria such as *Bifidobacterium* but also inhibit the growth of pathogenic bacteria, while improving the intestinal mucosal barrier function [17,64,66,67]. The aforementioned evidence indicate that variation in commercial formulated diets, dominant seed-based rations, supplementation of fruits and vegetables, actual dietary fiber and fat content, as well as husbandry feeding practices, may contribute to directly shape the diversity and functional characteristics of parrot gut microbiota.

#### 3.3.4. Influence of Reproductive Cycle and Hormonal Changes

Fluctuations in hormone levels during the avian reproductive cycle are also critical physiological factors shaping the composition of gut microbiota [68,69]. Female birds entering the reproductive period often experience mild gastrointestinal disturbances, such as soft stools, due to increased intestinal sensitivity caused by hormonal changes. Furthermore, it is likely that birds cyclically acquire new bacteria during the breeding season, contributing to the phylogenetic diversity, richness, and community composition of their overall microbiome. These microbial characteristics tend to increase with the active reproductive condition of the birds [68]. In female Phayre’s leaf monkeys (*Trachypithecus phayrei*) reproductive hormones, specifically progestagens, contribute to the shifts in the gut microbiome during the reproductive cycle [70]. The proportion of Firmicutes can rise to 45% during the breeding season, and this type of microbiota can provide the energy required for reproduction to female animals through efficient metabolism. In the non-breeding season, Proteobacteria become the dominant microbiota (accounting for 52%), primarily responsible for maintaining intestinal immune homeostasis [70]. It is worth noting that this “reproductive cycle-dependent microbiota fluctuation” is significantly weakened in inbred individuals, and abnormal microbiota fluctuation may further affect the reproductive performance and the health of their offspring. Mechanistically, changes in hormone levels, such as cortisol during the reproductive period, can “selectively screen” the gut microbiota by altering intestinal permeability and mucus secretion, ultimately forming a microbiota structure adapted to reproductive needs. Relevant studies on insect models have also provided supplementary evidence for this mechanism: the reproductive period microbiota can affect yolk synthesis by regulating the tryptophan metabolism process, directly highlighting the critical contribution of gut microbiota to host reproductive output [71]. Accordingly, these studies may serve as comparative evidence that hormonal fluctuations during the reproductive period can influence the gut microbiota to match the physiological needs of different reproductive stages. For parrots, there is a lack of direct evidence.

**Table 3:** Factors affecting the gut microbiota of parrots.

Category	Subcategory	Effects	Mechanisms	References
Age and growth stage	Young parrots	High consistency in Firmicutes/γ-Proteobacteria ratio, unstable microbiota structure, high sensitivity to external environment.	Immature intestinal microenvironment and immune system lead to conserved microbiota structure.	[41,53,55,57,58]
	Adult parrots	Significant individual differences in microbiota, decreased diversity but enhanced functional specialization (e.g., energy metabolism).	Mature intestinal microenvironment and differentiated immune system exert personalized screening on microbiota.	
Environmental and management factors	Contamination of the water supply system	Contaminated drinking water (Salmonella, *E. coli*) causes microbiota imbalance.	Pathogens from contaminated water colonize the intestine and disrupt microbial balance.	[41,59,60,61,62]
	Cage cleaning	Inadequate cage cleaning increases parasite infection risk, exacerbating microbiota disorders.	Parasites disturb the “microbiota-host immunity” relationship.	
Dietary and nutritional factors	Long-term high-fat/high-sugar seed diets	Reduced microbiota diversity, increased Proteobacteria (Enterobacteriaceae) proportion, induced chronic intestinal inflammation.	High-fat/sugar diets alter microbial composition and trigger inflammatory responses.	[54,63,64,65,66,67]
	Diverse diets	Improved microbiota diversity/richness, promoted SCFA-producing bacteria (e.g., Ruminococcus).	Diverse diets provide varied nutrients for microbial proliferation, and SCFAs protect intestinal health.	
	Functional feed components (probiotics, prebiotics, plant extracts)	Promoted beneficial bacteria (e.g., *Bifidobacterium*), inhibited pathogens, enhanced intestinal mucosal barrier.	Functional components modulate microbial communities by promoting beneficial bacteria and inhibiting pathogens, and strengthen the intestinal barrier.	
Reproductive cycle and hormones	Breeding season	The relative abundance of Firmicutes increases (this taxon may supply energy through efficient metabolic pathways that potentially support reproduction).	Hormonal changes (e.g., cortisol) during reproduction alter intestinal permeability and mucus secretion, selectively screening microbiota	[68,70,71]
	Non-breeding season	Proteobacteria become dominant, potentially modulating intestinal immune homeostasis.	Reproductive microbiota regulates tryptophan metabolism to affect yolk synthesis	
	Inbred individuals	Weakened microbiota fluctuation, which may affect reproductive performance and offspring health.	Inbreeding reduces microbiota adaptability to reproductive cycle hormonal changes	

### 3.4. Mechanism Between Gut Microbiota and Host Health

#### 3.4.1. Nutrient Metabolism and Energy Conversion

Gut microbiota plays a pivotal role in nutrient metabolism. It can metabolize complex carbohydrates, such as dietary fiber, which the host cannot digest directly, thereby producing metabolites such as SCFAs, mainly including acetate, propionate, and butyrate [72]. Evidence from other animal models indicates that these products can not only be utilized by the host but also participate in regulating the host’s energy balance [73,74,75]. Specifically, after absorption by intestinal epithelial cells, SCFAs can promote hepatic gluconeogenesis and fat metabolism; they can also regulate the host’s appetite and energy expenditure by activating G protein-coupled receptors such as GPR41 and GPR43 [10,75,76]. Additionally, gut microbiota is deeply involved in protein metabolism and amino acid conversion processes. Substances such as indole derivatives produced by their metabolism can influence intestinal epithelial cell function by activating the aryl hydrocarbon receptor (AhR) pathway, which is of great significance for the energy supply and efficient utilization of nutrients in parrots [75]. At the same time, B vitamins such as vitamin B12 and folic acid secreted by probiotics can promote carbohydrate metabolism and effectively improve the energy conversion efficiency of feed [73,74]. Importantly, when gut microbiota is imbalanced, SCFA production is significantly reduced, leading to impaired host energy acquisition and possibly inducing metabolic diseases such as obesity [76].

#### 3.4.2. Immune Regulation and Disease Resistance

Gut microbiota can regulate the immune function through various pathways to enhance disease resistance. Beneficial microbiota, including *Lactobacillus* strains, may help inhibit the proliferation of pathogenic bacteria like *Salmonella* and reduce the risk of infection by competing for nutrients and intestinal colonization sites, as well as secreting antibacterial peptides [77,78]. At the same time, SCFAs produced by beneficial microbiota nourish intestinal epithelial cells and promote the expression of tight junction proteins such as ZO-1, Occludin, and Claudin-1, thereby reducing intestinal permeability and preventing pathogen translocation [77,79]. At the level of immune cell regulation, microbiota metabolites can activate the retinoic acid metabolism process of dendritic cells (DCs), inducing B cells to differentiate into secretory immunoglobulin A-positive (sIgA^+^) plasma cells. The sIgA produced by these plasma cells can neutralize pathogens and maintain gut microbiota homeostasis [77,78]. Furthermore, SCFAs can promote the differentiation of regulatory T cells (Treg) by inhibiting histone deacetylase (HDAC). This action helps avoid the imbalance of Th1/Th2 immune cell polarization, thereby alleviating intestinal inflammatory responses and improving the overall disease resistance of parrots [77]. Conversely, studies in other avian models suggest that altered gut microbial composition is linked to reduced avian immunity, rendering birds more susceptible to pathogen infection [11]. Therefore, prebiotics such as fucoidan and inulin can be strategically incorporated into the diet; this can increase the abundance of intestinal beneficial bacteria such as *Lactobacillus* and *Bifidobacterium* and enhance SCFA production, which may contribute to restore intestinal barrier function [8,77]. However, research on parrots remains limited, and further studies including metagenomic, metabolomic, and functional validation are needed to definitively establish how gut microbiota influences host health.

#### 3.4.3. Microbiota Imbalance and Disease Occurrence

Gut microbiota perturbation is closely related to a series of health problems in birds, such as indigestion, diarrhea, and decreased immunity. When the balanced state of the gut microbiota is disrupted, it creates a predisposition to the excessive proliferation of *Candida* and *Salmonella* infections, which may be associated with symptoms such as intestinal mucosal erosion and bloody stools [77,80,81]. Additionally, coccidiosis infection is also closely associated with gut microbiota perturbationis: coccidia can damage intestinal epithelial cells, further exacerbating the dysregulation of the gut microbiota–immune axis. This leads to intestinal erosion and hemorrhage, inducing symptoms such as bloody stools and posing a serious threat to the animal’s health [13,80,82]. It can be seen that a range of important parrot diseases, including PaBV-induced proventricular dilatation disease, parasitic enteric infections, and secondary bacterial intestinal disorders, are closely intertwined with gut-health impairment and microbiota perturbationis. Thus, maintaining the stable balance of gut microbiota is an important component of intestinal health in preventing intestinal diseases.

## 4. Key Factors Affecting the Intestinal Health of Parrots

**Figure 2:**
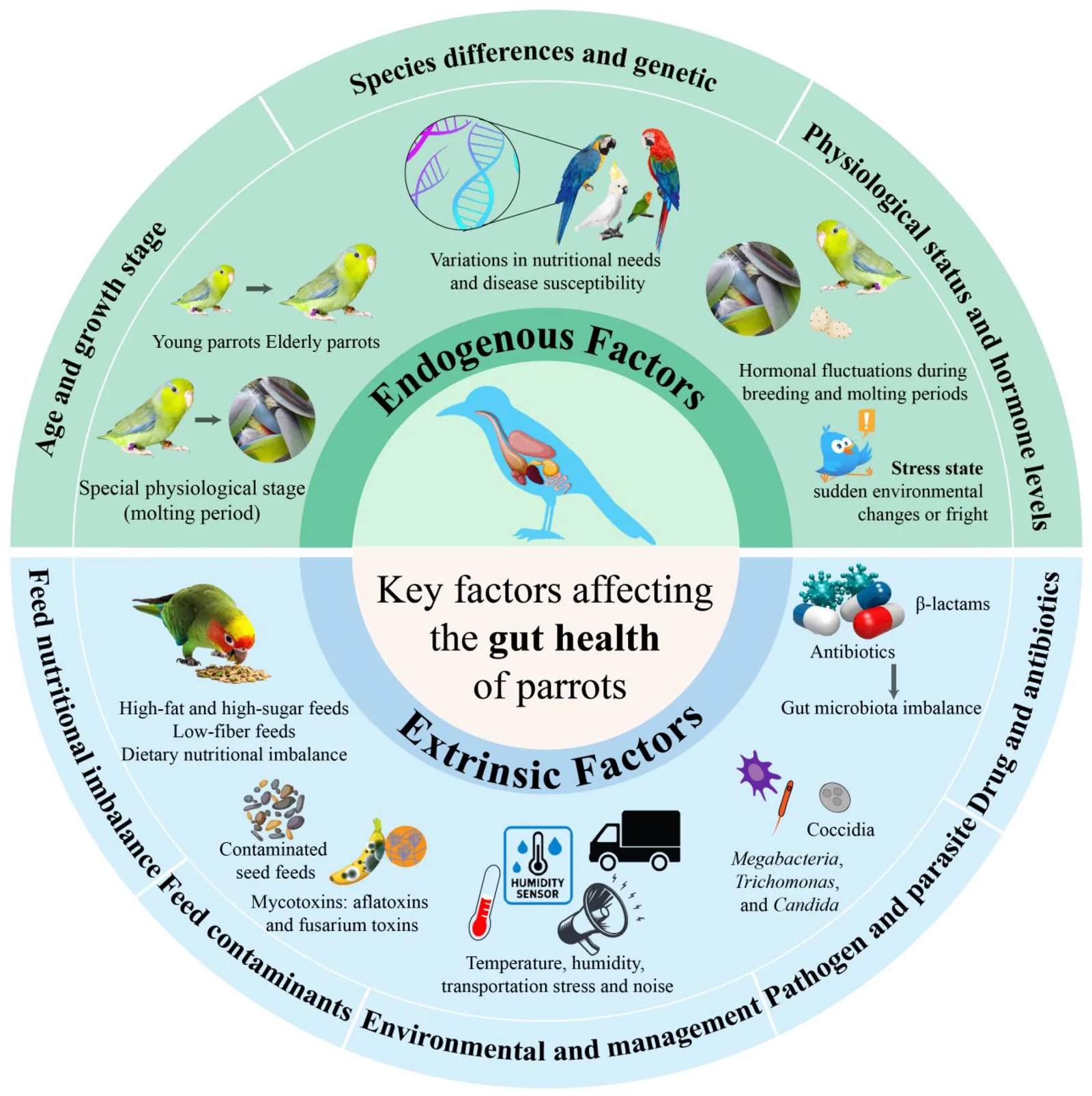
Key factors affecting the intestinal health of parrots.

At present, there are more studies related to the gut health of poultry, while there are relatively fewer studies on the gut health of parrots. Therefore, most of the content is organized based on the research on poultry. The intestinal health of birds is governed by a complex interplay of endogenous and extrinsic factors. Endogenous determinants, such as age, growth stage, species, genetics, and hormonal status, are pivotal. Juvenile, geriatric, molting, and breeding parrots face heightened risks due to microbiota instability, hormonal fluctuations, and compromised intestinal barriers. Externally, factors including unbalanced diets, mycotoxin exposure, environmental stress, pathogens, parasites, and inappropriate antibiotic use disrupt gut microbiota homeostasis and damage intestinal architecture. Collectively, these factors impair digestive efficiency, weaken immunity, and increase disease susceptibility (Figure 2 and Table 4).

### 4.1. Endogenous Factors

#### 4.1.1. Influence of Age and Growth Stage

The intestinal health status of parrots is significantly regulated by age and growth stage. Juvenile and geriatric parrots exhibit increased vulnerability regarding intestinal health, while the molting period, as a distinct physiological stage, presents unique intestinal health challenges [62]. In Juvenile parrots, the digestive system is not yet fully developed; it is characterized by low activity of key digestive enzymes, such as protease and amylase, and the gut microbiota colonization has not yet established a stable equilibrium. These factors collectively result in low nutrient absorption efficiency and significantly increased susceptibility to pathogenic microorganisms in juvenile birds [63,83,84,96,97]. Conversely, geriatric parrots experience physiological decline, leading to reduced regenerative capacity of intestinal epithelial cells, decreased digestive enzyme secretion, and diminished gut microbiota diversity. These issues further impair digestive function and weaken the intestinal barrier, increasing the risk of intestinal diseases [62,84,97]. The molting period (typically occurring from March to April annually), as a special physiological stage for birds, is accompanied by drastic fluctuations in hormone levels, such as thyroxine and cortisol, which exert multiple adverse effects on intestinal health. For instance, these hormones can suppress digestive enzyme activity. Additionally, they decrease intestinal mucus secretion and inhibit the expression of tight junction proteins, leading to increased intestinal permeability and a significantly elevated risk of inflammation. Note that this magnitude of enzyme-suppression effect comes from non-psittacine avian models and lacks direct quantitative measurements in parrots [51,85,98].

Targeted nutritional regulation strategies should be formulated based on the intestinal health characteristics of different growth stages. Juvenile parrots require supplementation with digestible proteins and prebiotics to support the stable establishment of gut microbiota. Geriatric parrots should receive increased intake of antioxidants, such as vitamin E and selenium (Se), to alleviate intestinal oxidative damage. During the molting period, it is necessary to strengthen the supply of essential fatty acids and B vitamins to meet the special metabolic demands of this stage [51,84,96,97,98].

#### 4.1.2. Species Differences and Genetic Factors

Different parrot species exhibit inherent differences in intestinal structure and function, which directly result in significant variations in their nutritional requirements and susceptibility to intestinal diseases. Medium and large parrot species, such as cockatiels, possess longer intestinal tracts, resulting in relatively longer food retention time. If maintained on a long-term single diet, these birds are prone to deficiencies in B vitamins and fat-soluble vitamins. These deficiencies can further lead to impaired intestinal mucosal repair, resulting in suppressed immune function and an increased risk of intestinal infections [56,86,99]. Small parrot species such as budgerigars, with shorter intestinal lengths and lower microbial abundance, have significantly weaker resistance to parasites such as *Giardia* and opportunistic pathogens like *E. coli*, rendering them more susceptible to intestinal infections [56,100]. In addition to species differences, inbreeding among genetic factors also adversely affects parrot intestinal health. Inbreeding reduces gut microbiota α-diversity, disrupts in the ratio of Firmicutes to Bacteroidetes, and decreases genetic heterogeneity. This impairment compromises the ability of gut microbiota to metabolize SCFAs and reduces intestinal barrier function and immune response levels [56,87,100]. Furthermore, genetic factors related to inbreeding can regulate the expression of tight junction proteins, such as occludin and ZO-1, in the intestinal epithelial cells, affecting the efficiency of intestinal pathogen clearance [87], ultimately leading to a general decline in parrot resistance to environmental changes and pathogen invasion.

#### 4.1.3. Physiological Status and Hormone Levels

Hormonal changes in parrots during the breeding period and stress states directly disrupt intestinal homeostasis and have a significant impact on intestinal health. Additionally, hormonal fluctuations during the molting period indirectly affect intestinal function. During the breeding period, estrogen and progesterone levels in female birds increase sharply. These hormones can promote the expression of pro-inflammatory factors, such as IL-1β and TNF-α, by activating the NF-κB signaling pathway. The abnormal increase in pro-inflammatory factors leads to increased intestinal sensitivity and digestive disorders, making female birds prone to indigestion during the breeding period [88]. When parrots experience stress, such as sudden environmental changes or fright, cortisol concentration rises rapidly [101]. This hormone damages intestinal health in multiple ways: inhibiting mucin secretion by goblet cells, reducing the abundance of beneficial bacteria such as *Lactobacillus*, and disrupting the tight junction structure of intestinal epithelium, ultimately inducing gut microbiota translocation and systemic inflammation [51,85]. Furthermore, hormonal fluctuations in birds during the molting period can reduce intestinal nutrient absorption efficiency by affecting the transcription of digestive enzyme genes, such as trypsinogen, and altering intestinal peristalsis rhythm. Based on the intestinal health needs under different physiological states, targeted nutritional regulation is required. During the breeding period, ω-3 fatty acids should be supplemented to alleviate intestinal inflammation through their anti-inflammatory properties. During the stress period, the addition of probiotics (e.g., *Bacillus*) and glutamine is necessary to regulate gut microbiota balance and repair intestinal epithelial cells, thereby jointly restoring intestinal barrier function [88].

### 4.2. Extrinsic Factors

#### 4.2.1. Feed Factors and Nutritional Imbalance

Feed quality is a core determinant of the intestinal health status. Long-term exclusive feeding of high-fat, high-sugar diets, such as sunflower seeds, can lead to vitamin A deficiency, thereby causing damage to the digestive tract mucosa and significantly impairing intestinal immunity [5,50,89]. Nutritional imbalance also directly alters the composition of gut microbiota. Low-fiber feeds reduce the population of beneficial bacteria such as *Lactobacillus* and *Bifidobacterium*, while promoting the abundance of opportunistic pathogens, ultimately weakening intestinal barrier function [5,50,89,102]. Additionally, imbalanced nutrient proportions can induce intestinal problems. Excessive protein intake increases the metabolic burden on the liver and kidneys, often causing diarrhea, while calcium deficiency interferes with the normal contraction function of intestinal smooth muscle. Collectively, these disturbances cause digestive dysfunction in birds and significantly increase the risk of diarrhea [50,89]. At the same time, insufficient fiber content in the feed slows intestinal peristalsis, increasing the probability of constipation.

#### 4.2.2. Hazards of Feed Contaminants and Mycotoxins

Mycotoxins, such as aflatoxins and fusarium toxins, enter birds’ bodies mainly through contaminated low-quality seed feeds, causing dual damage to intestinal health. First, these toxins directly disrupt the tight junction structure between intestinal epithelial cells, increase intestinal mucosal permeability, and induce “leaky gut” phenomenon, leading to impaired intestinal barrier function. Second, toxins inhibit the growth of beneficial bacteria such as *Lactobacillus*, promote the proliferation of pathogenic bacteria, significantly reduce the α-diversity of gut microbiota, and ultimately result in an altered microbial composition [43,90,91,95]. This dual-hit effect may contribute to liver damage and digestive dysfunction [40,103]. Notably, birds are significantly more sensitive to aflatoxins than other species due to their short digestive systems and high absorption efficiency; consequently, mycotoxin contamination poses a particularly prominent threat to bird intestinal health [43,90].

#### 4.2.3. Environmental Stress and Management Factors

The environment plays a crucial role in shaping an animal’s response to challenges such as disease, stress, and pathogens. Environmental factors, including sudden changes in temperature and humidity and transportation stress, directly impact animal well-being [104]. When birds are under stress, the intestinal pH value rise. This inhibits the growth of acid-tolerant beneficial bacteria, such as *Lactobacillus*, and creates favorable conditions for the colonization of opportunistic pathogens, such as *Escherichia coli*. Ultimately, this leads to gut microbiota imbalance, specifically manifested as loose stools, loss of appetite, and dull plumage [50,89]. Additionally, inappropriate temperature and humidity indirectly affect intestinal health: high temperature causes damage to the intestines, liver and kidneys (as observed in broiler chickens); excessively high humidity promotes the proliferation of pathogens such as *Trichomonas*; and excessively low humidity can compromise the bird’s skin barrier, leading to decreased immunity [89,92,105,106]. Environmental stress, such as noise, can affect gut microbiota diversity and structure by increasing stress hormone levels (e.g., cortisol) [52]. Furthermore, poor cage hygiene increases the risk of birds being exposed to pathogens, ultimately forming a vicious cycle of “environmental stress–microbiota disorder–pathogen infection” that continuously threatens intestinal health [89,92].

#### 4.2.4. Pathogen Infection and Parasite Influence

Intestinal pathogen infection is a significant inducement for parrot intestinal health problems. Common enteric pathogens include *Megabacteria*, *Trichomonas*, and *Candida*, each with different pathogenic mechanisms and hazards. *Megabacteria* mainly infect the proventricular isthmus of parrots, leading to chronic wasting in infected individuals. *Trichomonas* mainly infects the upper digestive tract, causing oral ulcers and erosive lesions. *Candida*, as an opportunistic pathogen, proliferates excessively when parrot immunity is low, thereby triggering infection [89,93,94]. In addition to pathogens, parasite infections, such as coccidia, also cause severe damage to the intestines, directly leading to symptoms including intestinal mucosal erosion and bloody stools, and significantly increasing the mortality rate of infected parrots [40,82,93]. Notably, parasites can also increase host susceptibility to secondary bacterial infections by altering gut microbiota niches—such as by reducing the thickness of the protective mucus layer—thereby exacerbating intestinal damage [89,93,94].

#### 4.2.5. Influence of Drug Use and Antibiotics

Indiscriminate antibiotic use is a key factor disrupting the balance of bird gut microbiota and inducing the proliferation of drug-resistant bacteria. Broad-spectrum antibiotics, particularly β-lactams, indiscriminately deplete beneficial bacteria in the intestines, which can be associated with massive proliferation of drug-resistant bacteria such as *Clostridium difficile*. Meanwhile, reduced abundance of beneficial bacteria may lower the production of anti-inflammatory metabolites such as SCFAs, potentially contributing to gut microbiota perturbationis [74,95]. This imbalance further leads to symptoms such as diarrhea, anorexia, and weakness in birds, accompanied by markedly suppressed immunity. Moreover, intestinal barrier function is impaired, specifically manifested as decreased number of goblet cells, insufficient mucus secretion, and increased levels of inflammatory markers [74,94,95]. Studies have shown that timely supplementation of probiotics, such as *Lactobacillus,* can effectively accelerate intestinal mucosal repair [77], and that supplementing probiotics 48 h after the use of antibacterial drugs can significantly restore gut microbiota diversity and reduce the risk of secondary infections [74,77,107,108]. Therefore, the timely supplementation of probiotics following antibiotic administration may be a key measure to maintain parrot intestinal health.

## 5. Nutritional Regulation Strategies for Parrot Intestinal Health

Nutritional regulation plays a significant role in managing the intestinal health of parrots. Common nutritional regulation methods include macronutrients, micronutrients, probiotics and prebiotics, and plant extract, all of which exert different effects on the modulation of intestinal health (Table 5).

### 5.1. Regulation of Macronutrients

Given parrots’ unique anatomical traits including degenerated ceca, gallbladder absence and short intestinal tract, their nutritional requirements differ substantially from those of other poultry species. Nutritional regulation for parrots should match these species-specific physiological features, and results from poultry studies can only serve as indirect references.

#### 5.1.1. Protein Requirements and Optimization

As a core nutrient for parrot growth and development, intestinal protein absorption efficiency directly determines overall health. Due to differences in parrot species and growth stages, their protein requirements vary significantly. Both rapidly growing chicks and breeding females, for instance, have elevated needs to support their specific physiology. Studies conducted in non-psittacine animals have shown that specific probiotics such as *Lactobacillus* spp. may promote the uptake of peptides and amino acids by upregulating the expression levels of intestinal amino acid transporters (LAT1, LAT2, and PepT2), which may improve protein-absorption efficiency [109,110,111]. The mechanism involves probiotic modulation of the gut microbiota, which enhances the activity of amino acid transporters, and thereby optimizes the utilization of protein substrates in the intestine [111,112]. In addition, protein hydrolysates play a key role in optimizing protein utilization and can activate the TOR signaling pathway and inhibit the amino acid response (AAR), reducing the oxidative decomposition of amino acids and further improving protein utilization efficiency [138,139,140]. However, a higher protein intake is not always better. Excessive protein increases the metabolic burden on the liver and kidneys of parrots, potentially triggering digestive disorders and intestinal inflammatory responses. Therefore, dietary protein levels must be carefully regulated [25,141].

#### 5.1.2. Fat Metabolism and Supply Strategies

Due to the physiological absence of a gallbladder, parrots have a limited capacity for fat digestion [113]. Consequently, it is advisable to avoid high-fat foods, such as egg-based feeds or millet mixed with egg, during breeding. If the fat content in the feed is too high, it may disrupt the homeostasis of gut microbiota, specifically manifested by the excessive enrichment of Firmicutes, which in turn increases the production of secondary bile acids, activates FXR receptors, potentially promotes intestinal lipid deposition, and raises the risk of acute enteritis [110,112,114]. According to the fat metabolism characteristics of different parrot species, dietary fat levels and fatty acid profiles may be adjusted in a targeted manner. Regarding fat source selection, easily digestible fats such as vegetable oils may serve as suitable candidates. Meanwhile, lipase preparations could be supplemented to mitigate intestinal stress induced by high-fat loads by improving fat digestibility and modulating lipolysis [110,142,143]. The studies further suggest a close interaction between fat sources and gut microbiota, which may strongly influence the profile of intestinal secondary metabolites [114,142]. Therefore, this gallbladder-deficient physiological trait and the involvement of gut microbiota in fat metabolism regulation should be fully considered when designing fat-related nutritional schemes for captive parrots.

#### 5.1.3. Configuration of Carbohydrates and Fiber

Owing to the complete degeneration of ceca in parrots, their capacity for microbial-driven fiber fermentation is significantly weaker than poultry with functional ceca. An appropriate fiber ratio is therefore one of the key factors maintaining parrot intestinal health. Insufficient fiber content in the feed will slow intestinal motility and increase the risk of constipation, whereas excessive fiber content will interfere with the absorption and utilization of other nutrients such as proteins and fats. Studies have shown that when the fiber level in the feed is controlled within the appropriate range of 5–7%, it can effectively promote the production of SCFAs, such as butyrate, in the intestine. SCFAs can enhance intestinal barrier function and upregulate the expression levels of tight junction proteins (ZO-1, Occludin), thereby improving intestinal defense capacity [25,115]. Given the anatomical degeneration of the ceca in parrots, carbohydrate-source selection requires careful optimization. Easily digestible carbohydrates, including waxy corn starch, could be considered as potential options. These carbohydrates can not only reduce amino acid catabolism for energy but also promote muscle protein synthesis. Simultaneously, enzyme preparations such as non-starch polysaccharide enzymes (e.g., xylanase) can be appropriately added to break down complex carbohydrate structures and improve carbohydrate utilization efficiency [25,116]. In addition, relevant studies have found that some probiotic strains possess a unique ability to decompose plant fiber and adding such probiotics to the feed can further improve the digestion and utilization of fibrous ingredients by parrots [25,115].

### 5.2. Regulation of Micronutrients

Most of the described micronutrient-related intervention strategies are extrapolated from other avian and mammalian models. They represent potential approaches rather than validated routine schemes for parrots, and direct experimental verification in psittacine birds is still needed.

#### 5.2.1. Vitamin Requirements

Vitamin A (VA) is essential for the health of the digestive tract mucosa, and its active metabolite retinoic acid (RA) may directly modulate the integrity of the intestinal barrier. When VA is deficient, noticeable lesions will occur in the intestinal mucosa, including a decrease in the number of goblet cells, thinning of the mucus layer, and downregulation of the expression level of tight junction proteins (such as ZO-2), potentially leading to elevated intestinal permeability and a ‘leaky gut’ phenotype [117,118]. The mechanism involves RA activating the Toll-like receptor 4 (TLR4) signaling pathway by binding to retinoic acid receptor β (RARβ), thereby promoting the synthesis of tight junction proteins and strengthening intestinal physical barrier function [117]. At the same time, VA can maintain the immune homeostasis of the intestinal mucosa by regulating the homing process of CD4^+^T cells in the intestinal lamina propria and the differentiation of IgA^+^B cells [144]. Thus, VA deficiency may simultaneously compromise intestinal mucosal integrity and decreases immunity. Notably, certain gut microbiota such as *Lactobacillus* and *Bifidobacterium* can metabolize VA to produce RA, establishing a positive feedback loop of “microbiota–VA–immunity” to further strengthen the intestinal health regulation effect [81,145].

B vitamins are crucial for energy metabolism and the regulation of intestinal function. Their supply should be appropriately increased, especially when parrots are under stress conditions. Additionally, probiotics can synthesize B vitamins to promote carbohydrate metabolism and improve feed utilization. However, there are no reports on specific studies on B vitamins in parrots to date. Both vitamin E (VE) and vitamin C (VC) are antioxidants, reducing intestinal epithelial cell damage by scavenging free radicals in the intestine. Relevant experiments have confirmed that the combination of VE and selenium (Se) can significantly increase the activity of glutathione peroxidase (GPX), reduce the levels of oxidative damage markers such as malondialdehyde (MDA), and may alleviate heat-stress-triggered increases in intestinal permeability [119]. VC can strengthen intestinal barrier function by regulating gut microbiota composition, such as increasing *Lactobacillus* abundance and inhibiting the release of pro-inflammatory factors like TNF-α and IL-6 [74,146]. Furthermore, the combined application of VE and VC in the laying hen heat stress model can significantly improve feed utilization, increase eggshell thickness, and enhance antioxidant enzyme activity, fully confirming their synergistic effect [120].

#### 5.2.2. Mineral Balance

Macrominerals such as calcium and phosphorus are essential for animal bone development and are crucial for maintaining intestinal muscle function [95,121]. However, excessive calcium supply can lead to mineral deposition, appearing as a white, radiopaque layer surrounding the liver on radiographs. Female birds, particularly during the laying period, are prone to calcium deficiency, which in turn causes impaired intestinal peristalsis and affects normal digestive function. Trace elements such as selenium (Se) and zinc (Zn) enhance intestinal antioxidant capacity and improve intestinal barrier function [131]. As the important component of GPX, Se can alleviate oxidative damage by reducing the ratio of oxidized glutathione (GSSG) to reduced glutathione (GSH) in intestinal tissue; the combined use of Se and VE can significantly improve intestinal permeability under heat stress [119]. Zn can promote the repair of intestinal epithelial cells and enhance the expression of antibacterial peptides. Its deficiency aggravates intestinal mucosal ulcers and inflammatory responses. Meanwhile, when supplementing minerals, it is critical to maintain balanced mineral ratios to avoid antagonism caused by imbalanced proportions, such as high calcium inhibiting Zn absorption, ensuring that minerals function optimally.

### 5.3. Application of Probiotics and Prebiotics

It should be noted that most of the available evidence regarding gut-modulating effects of probiotics and nutritional interventions is obtained from commercial poultry. Given the physiological differences between parrots and conventional poultry, these findings can only serve as indirect references for parrot healthy breeding, and these proposed intervention strategies should be regarded as potential candidates awaiting validation in psittacine species.

#### 5.3.1. Screening of Novel Probiotic Strains

Based on the research strategies of other poultry species, the screening of parrot-specific probiotics should focus on acid resistance and bile salt resistance. Common high-quality strains include *Saccharomyces boulardii* (CNCM I-1079) and *Bacillus subtilis* (such as BCM-7 type) that exhibit high tolerance to gastric acid and bile salts in the digestive tract by forming spores or special cell wall structures. The latest research data indicate that *Bacillus subtilis* BCM-7 possesses particularly outstanding acid resistance, with a spore survival rate up to 98% in a gastric acid environment; these acid-challenge data are obtained from in-vitro laboratory assays rather than parrot in-vivo tests. At the same time, the extracellular matrix (ECM) secreted by this strain can encapsulate other probiotics such as *Lactobacillus* to jointly resist digestive stress [123,124]. In addition, acid-resistant strains such as *Lactobacillus plantarum* Lp-22 may serve as potential candidates. These strains can colonize the intestinal wall through strong adhesion to form a biological barrier. The threshold of >5 billion CFU per gram represents a reference value derived from non-psittacine experimental systems; such dosage benchmarks have not been validated for parrots [124,125,147]. The *Saccharomyces cerevisiae* SC-27 strain offers unique advantages, as the cellulase it secretes can decompose plant fiber, further improving the nutrient utilization [124,126].

#### 5.3.2. Compound Probiotics

A compound probiotic system consisting of *Saccharomyces boulardii* and *Lactobacillus* can achieve a synergistic effect of a dual-layer defense. *Saccharomyces boulardii* inhibits the proliferation of harmful bacteria such as *Salmonella* by secreting antibacterial peptides, while *Lactobacillus* regulates intestinal pH by producing SCFAs, further inhibiting pathogen growth and significantly reducing the risk of intestinal infection [17,126,127]. The combined application of *Bacillus subtilis* and *Saccharomyces cerevisiae* also has synergistic value. *Bacillus subtilis* can synthesize B vitamins and enhance intestinal barrier function, while *Saccharomyces cerevisiae* provides metabolic substrates for gut microbiota by decomposing fiber, thereby improving overall digestive efficiency [124,127,128]. Additionally, the ECM of *Bacillus subtilis* can physically embed *Lactobacillus*, thereby improving the survival rate of *Lactobacillus* during processing and storage [123,124]. In summary, compound-probiotic combinations may exert synergistic beneficial effects observed in non-psittacine models. These potential advantages have not been confirmed for parrots, and the performance of multi-strain formulations remains to be experimentally verified in psittacine birds.

#### 5.3.3. Probiotic Formulations

Currently, commercially available probiotics are mainly divided into three forms: powder, oil, and tablet. The characteristics of different formulations determine their specific applications. Powders use anhydrous glucose as a carrier and maintain a viability rate of up to 78% after 90 days of room-temperature storage. They blend uniformly into soft food, making them suitable for a daily feed addition to meet the regular intestinal health care needs of birds [125]. Oil-based formulations use oil to isolate moisture, making them suitable for short-term, high-frequency use; however, they have low stability during long-term storage and are thus primarily used for intestinal function regulation during the recovery period from illness [125]. Tablets have a relatively low survival rate of viable bacteria and are mainly used for emergency intervention when intestinal problems arise. To improve the stability of probiotic products, the newly developed microencapsulation technology can prolong strain viability under room-temperature storage under defined laboratory test conditions [124]. These storage-stability outcomes are obtained from experimental systems applied to species other than parrots and cannot be directly extrapolated to parrot-targeted preparations.

#### 5.3.4. Application of Prebiotics and Synbiotics

Prebiotics, which serve as a selective substrate for probiotics, synergize with them to form “synbiotics”, a formulation that regulates gut microbiota balance more efficiently than either component alone. Common prebiotics, such as oligosaccharides and inulin, can promote the growth of beneficial bacteria like *Lactobacillus* and *Bifidobacterium*, while inhibiting the growth of harmful bacteria [8]. Among them, the mechanism of inulin is relatively clear: by activating the *fos* and *pts* gene clusters of probiotics, it enhances the metabolic capacity of probiotics to ferment plant fibers [129,130]. Synbiotics combine prebiotics with probiotic strains to increase the production of SCFAs through synergistic effects, further strengthening the intestinal barrier and enhancing immune function [8,129]. According to the physiological characteristics of parrot intestines, developing specific synbiotic products may be a promising research direction in the future. However, relevant research in this field remains limited and warrants further investigation.

### 5.4. Others

#### 5.4.1. Application of Plant Extracts

Natural plant extracts are rich in various bioactive components such as polyphenols, flavonoids, and organic acids. They can improve intestinal health through multiple pathways, including antibacterial, anti-inflammatory, antioxidant, and gut microbiota modulation effects. Their core role is to change the composition and metabolites of the gut microbiota, thereby optimizing the intestinal microecological environment [10,17,131,148,149]. Studies have shown that blueberry extract can significantly increase SCFA production by enriching *Prevotella* and regulating the tryptophan metabolism pathway, which may support intestinal homeostasis [132]. In broiler breeding, tangerine peel extract can increase the number of *Bifidobacterium* and *Lactobacillus* in the intestine, reduce the abundance of *E. coli*, and increase concentrations of beneficial metabolites such as acetic acid and butyric acid, thereby enhancing immunoglobulin secretion [133]. Compound extracts of *Lonicera japonica* and *Scutellaria baicalensis* rely on active components such as chlorogenic acid and baicalin to reduce intestinal damage caused by *Salmonella* infection, downregulate the expression levels of pro-inflammatory factors such as TNF-α and IL-6, and increase the abundance of beneficial bacteria such as *Bacteroides* and *Faecalibacterium* [131,148]. Notably, Amazon parrots exhibit a natural behavior of pecking specific bark to regulate gut microbiota. The natural bacteriostatic components contained in such bark have similar mechanisms to bacteriophages. This species-specific feeding behavior further highlights the necessity of developing parrot-oriented functional additives rather than directly adopting additives optimized for poultry.

#### 5.4.2. Application of Bacteriophage Preparations

As a new biological control method, bacteriophage preparations have significant targeting advantages through their precise recognition and lysis ability for specific pathogenic bacteria (such as *E. coli*), thereby maintaining intestinal microecological balance without affecting beneficial microbiota such as *Lactobacillus* [134,135,150]. Practical data from Suzhou Rare Bird Breeding Base show that after applying bacteriophage preparations in chick breeding, the survival rate of chicks increased by 27%, and the disease recovery cycle was shortened to 5–7 days, demonstrating its practical application value. Regarding characteristics, bacteriophage preparations have high specificity and make it difficult for pathogenic bacteria to develop drug resistance; thus, they have great potential in reducing antibiotic use and promoting environmentally friendly farming practices [150]. For example, in broiler models, phage therapy can effectively reduce the load of pathogenic microorganisms in the intestine [134]. However, the large-scale production process of current bacteriophage preparations still requires optimization, and the safety of their long-term application warrants further verification. Moreover, existing studies mostly focus on poultry (e.g., broilers) and mammalian (e.g., pigs) models, and the application in parrot breeding is still in its initial stage. Future research should address key issues such as host specificity and long-term application effects.

#### 5.4.3. Exploration of Other Additives

In addition to the aforementioned plant extracts and bacteriophage preparations, novel additives such as enzyme preparations, organic acids, and SCFAs also show potential for regulating parrot intestinal health. The key role of enzyme preparations, such as amylase, protease, and cellulase, is to improve the digestibility of nutrients in the feed, reducing the digestive and metabolic burden on the intestine. They can indirectly maintain intestinal microecological homeostasis by reducing the residue of undigested substrates in the intestine and lowering the nutritional supply for harmful bacteria [28,83]. These effects have been verified in other species. For example, in dairy goat breeding, coix seed polyphenol extract can optimize the energy utilization efficiency by increasing the total amount of volatile fatty acids (VFAs) and the proportion of propionic acid in the rumen, while improving the contents of milk protein and lactose [136], offering insights for the application of enzyme preparations in parrot breeding. Organic acids, such as citric acid and propionic acid, optimize the gut microbiota structure by lowering the intestinal pH, creating an acidic environment unfavorable for the growth of harmful bacteria, and providing suitable conditions for the reproduction of beneficial bacteria [137]. SCFAs have more diverse functions, serving as the primary energy source for intestinal epithelial cells and exerting a protective effect on the intestine by regulating the immune function and intestinal barrier function [79]. For example, dietary supplementation with sodium acetate, a salt form of acetic acid which is a major cecal SCFA in rabbits, improved growth performance and intestinal health [151]. At present, the specific application effects, mechanisms, and optimal usage schemes of these novel additives in parrot breeding still need further experimental verification.

## 6. Conclusions

Gut microbiota is an important contributing factor for parrot intestinal health, mediating nutrient metabolism, immune modulation, and pathogen resistance. Furthermore, gut microbiota composition and function are shaped by diet, housing conditions, age, and reproductive status, making nutritional intervention a primary lever for maintaining gut homeostasis. Strategic formulation of macronutrients, targeted micronutrient supplementation, and rational use of probiotics and functional additives (e.g., plant extracts) represent promising intervention approaches for parrots. Their capacity to restore gut microbial balance, improve digestive-absorptive efficiency, and enhance host immunity still awaits direct empirical validation in psittacine species, including assessments of optimal formulation, suitable dosing, and safety profiles. The species-specific gastrointestinal anatomy and digestive physiology of parrots constitute the fundamental basis for formulating targeted nutritional regulation strategies, and direct extrapolation of poultry-derived nutritional schemes should be avoided. Despite notable progress in probiotic strain screening, compound preparation development, and gut microbiome profiling, critical knowledge gaps remain in parrots. The mechanistic basis of host microbiota crosstalk in parrots remains poorly resolved; parrot-specific functional additives suffer from low industrial translation; and a standardized, universally accepted intestinal-health evaluation framework is still lacking. In comparison, research on other poultry species in this field is relatively well-established, offering multiple aspects for reference.

Future research should prioritize interdisciplinary integration of microbiomics, nutritional science, and molecular biology to dissect host microbiota interaction mechanisms. Concurrently, efforts should focus on developing parrot-targeted functional products, establishing standardized intestinal-health assessment criteria, and advancing precision feeding and smart breeding technologies. Extending gut microbiota research to endangered parrot conservation will further bridge captive breeding management and wild population protection. Continued technological innovation and mechanistic deepening will ultimately underpin scientific intestinal health management strategies and promote the sustainable development of the parrot breeding industry.

## Figures and Tables

**Table 1:** Structure and function of the digestive system of parrots.

Structure	Key Features	Functions	Health Impacts	References
Crop	Specialized enlarged structure at the end of the esophagus with strong expansion capacity for temporary food storage. Secretes “crop milk” (fat-rich epithelial cells).	Enables rapid foraging and predator avoidance. Exclusive nutritional source for chicks but lacks carbohydrates and calcium.	Supplementing with probiotics may help improve crop health.	[14,19]
Muscular stomach (Gizzard)	Specialized muscle tissue with rough keratin-like membrane. Contains sand grains/pebbles for grinding. Powerful rhythmic contractions for mechanical digestion.	Compensates for “toothless” physiology Grinds hard seeds into fine particles. Determines nutrient digestibility and utilization.	The gizzard structure, thickness, and contractions are based mainly on the feed type. The grinding efficiency directly determines the utilization rate of nutrients in feed.	[20,21,22]
Small intestine	Intestinal length is equal to 2 to 3 times the body length (shorter than mammals).	Not clear.	Caused short chyme retention and limited nutrient absorption.	[22,23,24]
Rectum	Short and narrow, it cannot store a large amount of feces.	Excrete feces.	It jointly weakens the fermentation capacity of fibrous substances with cecal degeneration.	[22,23,24]
Cloaca	A shared channel for excretory and reproductive functions.	The terminal structure of the digestive tract, integrating the expulsion of feces and urine.	No direct digestive function, but can reflect the overall excretory status of the intestine.	/
Cecum	Completely degenerated ceca.	Limits fermentation of fibrous substances.	Results in lower intestinal microbiota diversity compared to birds with well-developed ceca, making the microbiota more susceptible to dietary disturbances.	[22,23,24]
Gallbladder	Lack of gallbladder.	Weak bile concentration.	Reduced tolerance to high-fat foods.	[25]
Liver and pancreas	/	Aid in digestion	/	/

**Table 2:** The influence of different feed on the digestive efficiency of parrots.

Feed Characteristics	Digestive Efficiency	Mechanisms	References
High protein/high starch	Significantly enhance	Stimulate digestive enzyme secretion.	[29,30]
High fiber	Reduce	Reliance on microbial fermentation, resulting in a slower rate.	[32]
Pre-gelatinization/Expansion treatment	Enhance	Increased surface area, facilitating enzymatic action.	[32]
Animal protein	High absorption rate	Easily decomposed by digestive enzymes.	[32]

**Table 4:** Factors affecting the intestinal health of parrots.

Category	Subcategory	Factor	Key Mechanisms	Health Impacts	References
Endogenous factors	Age and growth stage	Young parrots	Immature digestive system, low enzyme activity, unstable microbiota	Low nutrient absorption, increased pathogen susceptibility	[83,84,85]
		Elderly parrots	Reduced epithelial regeneration, decreased enzyme secretion	Impaired digestive function, weakened barrier	
		Molting period	Hormone fluctuations (thyroxine, cortisol)	Reduced enzyme activity, increased permeability	
	Species differences and genetics	Medium/large parrots	(e.g., cockatiels): longer intestines, longer food retention	B vitamin deficiencies, mucosal repair disorders	[56,86,87]
		Small parrots	(e.g., budgerigars): shorter intestines, low microbiota abundance	Higher parasite susceptibility (*Giardia*, *E. coli*)	
		Inbreeding	Reduced α-diversity, Firmicutes/Bacteroidetes imbalance	Reduced SCFA metabolism, lower barrier function	
	Physiological status and hormones	Breeding period	Estrogen/progesterone increase, NF-κB activation	Increased pro-inflammatory factors (IL-1β, TNF-α)	[51,85,88]
		Stress state	Cortisol elevation, reduced goblet cell function	Reduced Lactobacillus, microbiota translocation	
		Molting period	Hormone fluctuations affecting enzyme transcription	Reduced nutrient absorption, altered peristalsis	
Extrinsic factors	Feed factors and nutritional imbalance	High-fat/high-sugar feeds	Vitamin A deficiency	Mucosal damage, reduced immune function	[5,50,89]
		Low-fiber feeds	Reduced beneficial bacteria	Increased opportunistic pathogens	
		Excessive protein	Liver/kidney burden.	Diarrhea, digestive dysfunction.	
		Calcium deficiency	Smooth muscle dysfunction.	Constipation risk.	
	Feed contaminant	Mycotoxins	Mycotoxins (aflatoxins, fusarium toxins) from contaminated seeds disrupt tight junctions, inhibit beneficial bacteria, and reduce microbiota diversity.	“Leaky gut” phenomenon, impaired barrier function Microbiota imbalance; Liver damage, digestive dysfunction; Birds more sensitive to aflatoxins.	[43,90,91]
	Environmental stress	Temperature/humidity changes/transport stress	Altered intestinal pH, reduced *Lactobacillus*, increased *E. coli.*	Microbiota imbalance, loose stools, loss of appetite, dull feathers, and decreased immunity	[50,89,92]
		High humidity/low humidity	High humidity promotes Trichomonas; low humidity damages skin barrier/immunity.		
		Noise stress	Increased cortisol, changed microbiota structure.		
		Poor cage hygiene	Increases pathogen exposure.		
	Pathogen infection and parasite influence	Pathogens (*Megabacteria*, *Trichomonas*, *Candida*)	Megabacteria: proventricular isthmus infection. Trichomonas: upper digestive tract ulcers *Candida*: opportunistic overproliferation.	Chronic weight loss, ulcers Bloody stools, high mortality Secondary bacterial infections	[89,93,94]
		Parasites (coccidia)	Mucosal erosion, bloody stools.		
	Drug use and antibiotics	Improper use of antibiotics	β-lactam antibiotics: indiscriminate killing of beneficial bacteria Reduced SCFA production Impaired barrier function (goblet cell reduction)	Clostridium difficile proliferation Diarrhea, loss of appetite, weakness Reduced immunity, secondary infection risk	[74,77,95]

**Table 5:** Nutritional regulation strategies for parrot gut health.

Strategy Category	Regulatory Method	Evidence/Model	Key Mechanisms	Effects	References
Macronutrient regulation	Protein requirements and optimization	Poultry and mammalian models	Upregulation of amino acid transporters (LAT1, LAT2, PepT2) Activation of TOR signaling pathway Inhibition of amino acid response (AAR)	Increase in protein absorption rate Improved protein utilization efficiency Reduced metabolic burden on liver and kidneys	[109,110,111]
	Fat metabolism characteristics	In-vitro experimental evidence; Poultry model	Selection of easily digestible vegetable oils Addition of lipase preparations Targeted adjustment of fatty acid composition	Improved fat digestibility Maintenance of gut microbiota homeostasis Reduced risk of acute enteritis	[110,112,113,114]
	Carbohydrate and fiber configuration	Mammalian models	Appropriate dietary fiber inclusion Selection of easily digestible carbohydrates Addition of non-starch polysaccharide enzymes	Enhanced production of SCFAs (butyrate) Improved intestinal barrier function Upregulated tight junction proteins (ZO-1, Occludin)	[25,115,116]
Micronutrient regulation	Vitamin requirements	Poultry and mammalian models	Vitamin A (VA) for intestinal barrier integrity Vitamin B for energy metabolism Vitamin E and C for antioxidant protection	Maintenance of intestinal mucosa health Enhanced immune homeostasis Reduced oxidative damage	[117,118,119,120]
	Mineral balance	Poultry and mammalian models	Calcium and phosphorus for bone and intestinal muscle function Selenium (Se) and zinc (Zn) for antioxidant capacity Proportional balance between minerals	Improved intestinal antioxidant capacity Enhanced antibacterial peptide expression Prevention of mineral antagonism	[119,121,122]
Probiotics and prebiotics	Novel probiotic strains	In-vitro assays; poultry model	Acid resistance and bile salt resistance screening Spore formation and special cell wall structures Extracellular matrix (ECM) protection	High strain tolerance under gastric acid conditions Enhanced intestinal colonization capacity Improved fiber decomposition ability	[123,124,125]
	Compound probiotics	Poultry/mammalian models	Saccharomyces boulardii and Lactobacillus Bacillus subtilis and Saccharomyces cerevisiae ECM embedding technology	“Double defense lines” synergistic effect Inhibition of harmful bacteria proliferation Improved survival rate during processing	[126,127,128]
	Probiotic formulations	In-vitro & poultry models	Powder (retains viable bacteria under room-temperature storage) Oil (short-term high-frequency use) Tablet (emergency intervention) Microencapsulation technology	Extended viable-cell retention with microencapsulation Targeted application based on physiological state Improved stability during storage	[124,125]
	Prebiotics and synbiotics	Poultry and mammalian models	Oligosaccharides and inulin Activation of fos and pts gene clusters Synbiotic combinations (inulin and Lactobacillus)	Directional promotion of beneficial bacteria Increased SCFA production Alleviation of intestinal inflammation	[8,129,130]
Functional additives	Plant extracts	In-vitro assays/poultry/mammalian models; only field behavioral observation in Amazon parrots	Polyphenols, flavonoids, organic acids Regulation of gut microbiota composition Tryptophan metabolism pathway modulation	Enrichment of beneficial bacteria Increased SCFA production Enhanced immunoglobulin secretion	[17,131,132,133]
	Bacteriophage preparations	Poultry model/practical data from birds	Precise recognition of specific pathogens No impact on beneficial microbiota Targeted lysis of pathogenic bacteria	Improved chick survival Shortened disease recovery period Reduced antibiotic use	[134,135]
	Novel additives	Ruminant/poultry models	Enzyme preparations (amylase, protease) Organic acids (citric acid, propionic acid) Short-chain fatty acids (SCFAs)	Improved nutrient digestibility Optimized gut microbiota structure Enhanced antioxidant capacity	[136,137]

## Data Availability

No new data were created or analyzed in this study. Data sharing is not applicable to this article.
